# Deciphering Asthma Biomarkers with Protein Profiling Technology

**DOI:** 10.1155/2015/630637

**Published:** 2015-08-06

**Authors:** Zhizhou Kuang, Jarad J. Wilson, Shuhong Luo, Si-Wei Zhu, Ruo-Pan Huang

**Affiliations:** ^1^RayBiotech, Inc., Guangzhou 510600, China; ^2^RayBiotech, Inc., 3607 Parkway Lane, Norcross, GA 30092, USA; ^3^South China Biochip Research Center, Guangzhou 510600, China

## Abstract

Asthma is a chronic inflammatory disease of the airways, resulting in bronchial hyperresponsiveness with every allergen exposure. It is now clear that asthma is not a single disease, but rather a multifaceted syndrome that results from a variety of biologic mechanisms. Asthma is further problematic given that the disease consists of many variants, each with its own etiologic and pathophysiologic factors, including different cellular responses and inflammatory phenotypes. These facets make the rapid and accurate diagnosis (not to mention treatments) of asthma extremely difficult. Protein biomarkers can serve as powerful detection tools in both clinical and basic research applications. Recent endeavors from biomedical researchers have developed technical platforms, such as cytokine antibody arrays, that have been employed and used to further the global analysis of asthma biomarker studies. In this review, we discuss potential asthma biomarkers involved in the pathophysiologic process and eventual pathogenesis of asthma, how these biomarkers are being utilized, and how further testing methods might help improve the diagnosis and treatment strain that current asthma patients suffer.

## 1. Introduction 

### 1.1. Asthma

Asthma is an increasingly prevalent chronic disease of the conducting airways, affecting the lives of more than 300 million individuals worldwide. Common asthma symptoms include mucus overproduction, episodic airway obstruction, bronchial hyperreactivity (BHR), and reduced lung function. The disease has higher prevalence in wealthier communities compared to more impoverished areas, with 8–10% of adults and children being affected. This high disease prevalence leads to considerable morbidity and also causes significant economic burden on the affected families [1, 2]. This increased economic burden is estimated to cost more than $18 billion dollars annually in the US alone with regard to both direct medical bills and indirect productivity losses [3]. Given current trends in disease development, asthma could affect over 400 million people by 2025 if no further interventions are developed [1, 2].

Traditionally asthma is divided into two phenotypes based on the potential disease trigger, allergic asthma, or nonallergic asthma. Children are predominantly affected by allergic (extrinsic) asthma, while roughly 50% of adults have allergic asthma. Allergic asthma is typically characterized by high serum levels of IgE and predominantly type 2 CD4^+^ helper T cell (Th2) response [4]. This type of asthma begins generally with itching, shortness of breath, and inflammation of the lungs, often leading to rhinitis and worse asthma symptoms over the course of many allergen exposures [5]. Instead, nonallergic (intrinsic) asthma often arises later in life and is not generally associated with high levels of antiallergen IgE. Intrinsic asthma is more associated with disease or stress factors like obesity, anxiety, strenuous exercise, and even cold air. Given the noninflammatory nature of nonallergic asthma, it is generally much more difficult to treat, as steroids and other common therapies are not as effective as they are with asthma caused by environmental and immunological triggers [6].

Recent work over the last two decades has given the community increased understanding of the pathophysiological nature of asthma, leading to better classification of the disease into groups called endotypes [7]. These endotypes are characterized by multiple disease related factors including the clinical nature of disease, genetic susceptibility, environmental risk factors, and the age of disease onset [6]. The heterogeneity of asthma is therefore increasingly suggesting that asthma itself is more of a syndrome, rather than a single disease [8]. This heterogeneous disease type, however, makes early and accurate diagnosis of asthma increasingly difficult. Currently, the main means of disease confirmation relies on tests of overall pulmonary function (spirometry), yet this method is limited as there is a high degree of variability in the extent of airway obstruction within patient groups. Furthermore, measurements of lung function can reflect the pathological seriousness of disease but may not correspond precisely with different disease endotypes, creating only a partial snapshot of the patient's disease.

Treatment for asthma generally consists of systemic steroids, a method that has been employed for over 30 years. However, not all asthmatics respond to this generalized treatment, further highlighting the nature of such a heterogeneous disease. When considering the high cost and undesired side effects of steroidal therapies, there is a need for better endotype screening that affords better targeted treatments with more improved patient outcomes [9, 10]. Protein biomarkers have recently been explored and discovered for a number of diseases, and these markers have great advantages in clinical diagnoses, disease progression prediction, and personalized treatment. Given that these markers are often isolated and detected from noninvasive means (serum, plasma, urine, etc.) which limit the need for costly operations or surgeries, they could be performed alongside more historical tests providing more complete diagnostic profile of the patient. In fact, many biomarkers are already in use for the diagnosis and treatment of cardiovascular disease and certain cancers, as well as other complex disease states. With the progress the community has made over the past 25 years in understanding the cellular and molecular mechanisms of asthma disease, more asthma biomarkers could be just around the corner.

### 1.2. Biomarker

As with most diseases, the early, rapid, and accurate assessment of a patient's condition drastically improves long-term prognosis and/or survival. In order to provide a more accurate assessment of an individual's disease condition/state, a comprehensive and accurate medical diagnosis is needed, and asthma is no different. Such a specific diagnostic test for asthma should allow for a more accurately defined disease phenotype, with the ultimate goal of leading to more targeted therapies and accurate prognosis. With this goal in mind, biomarkers, defined as measurable indicators that can define a biological state such as a disease, infection, or environmental exposure, have attracted huge attention in both basic research and clinical studies. There are many types of biomarkers for a variety of disease pathways, some of which are even accepted by the medical community as standard means of disease detection. Other potential biomarkers, like microRNAs, are rapidly emerging as powerful markers for disease biology [11, 12]. Currently published biomarkers include microRNAs, small biological metabolites, presence/absence of cellular populations, or levels of various molecules or enzymes within a known sample.

Given their constant variation during disease development and progression, proteins provide a rich potential biomarker pool for disease identification [13]. Proteins can even provide multiple levels of disease information as they can be differentially expressed and processed during different disease states. By measuring changes in protein levels during any biological state, we can directly compare healthy states to various disease states, gathering a wealth of information on how these proteins change over the course of disease. This unique insight can show whether increased or decreased expression levels are present or whether there is a deviation of the norm which might indicate a divergence in the patient's condition. For example, C reactive protein (CRP) is a particularly valuable biomarker of inflammation which is overexpressed during inflammatory responses caused by an infection, by autoimmune diseases like lupus and RA, or in some cancers [14].

Secondly, differential protein processing, like changes to protein folding or protein glycosylation, can deviate from normal posttranslational modifications during disease. Tau aggregation into paired helical filaments in Alzheimer's disease (AD) and other tauopathies is associated with increased Tau protein phosphorylation in the brain [15]. Similarly, the development of antibodies to self-proteins, as seen with autoantibodies against rheumatoid factor (RF) in RA, can be another molecule to identify disease development and progression. Each of these can serve or have served as valuable markers of disease states and highlight the unique insight that proteins can provide to biological pathways and for diagnostic purposes.

The application of biomarkers is not applicable solely to the clinical diagnostic and prognostic fields but is also of great interest to the basic biology of disease, as these markers can also serve to evaluate drug toxicity and efficacy. Through 2012, the FDA has approved 23 protein tumor biomarkers for clinical use, of which 9 are used in different tumor diagnosis, 6 are for monitoring disease progression, and 5 are for prediction of response to therapies. 24 biomarkers were approved in 2007 for drug evaluation and research, including various effects related to toxicity and therapeutic effect (http://www.fda.org/). Biomarker approval has slowed in recent years, however, even while preclinical research and clinic trial studies continue to identify them and put them to use. Given this decline, it has been hypothesized that the “low hanging” biomarkers have already been found. These early biomarkers were easier to find as they were single protein associated with a given disease such as RF. Biomarkers to come will more likely be more complex, with multiple proteins or other molecules involved in various disease states, rather than a single marker. This multifactorial complexity will therefore require a new tool that can simultaneously measure multiple factors within a single sample.

### 1.3. Cytokines

Cytokines are among the most intensely studied protein families as they possess a myriad of local and systemic functions and also offer vast potential applications in biomedicine. In addition to playing critical roles in many normal cellular events, cytokines are also noted to be involved in the initiation, development, and progression of pathologies, from the innocuous to the life-threatening. For this reason, cytokines have long been explored and studied as potential disease biomarkers.

Cytokines are secreted proteins that generate or transmit cell-cell signals. These proteins include interferons, interleukins, chemokines, growth and differentiation factors, pro- and antiangiogenic factors, adipokines, adhesive molecules, and extracellular matrix proteins. Cytokines play a critical role in a number of cellular and bodily functions, from homeostasis, growth, and wound healing to inflammation, immunity, and angiogenesis [16–23]. Given their wide ranging role in the body, it is no surprise that cytokines have been associated with cancer, diabetes, and autoimmune and infectious diseases. It has been suggested that chronic inflammation plays a key role in neurodegenerative diseases, such as Alzheimer's and Parkinson's diseases, insulin resistance and type 2 diabetes, atherosclerosis, and other cardiovascular diseases [16, 24–27].

Typically, cytokines function as a complex network of specifically controlled signaling pathways. Their individual and even global signals are modulated by each other through cytokine specific receptors. Many cytokines are pleiotropic, meaning that a single cytokine may induce a wide range of effects in various cell and tissue types [28]. Interleukin-6 (IL-6), for example, acts as proinflammatory factor to stimulate macrophages and generate a Type 1 helper T cell response but can also act as an anti-inflammatory cytokine through its inhibitory effects on levels of TNF-alpha, IL-1, and its activation of IL-1ra and IL-10 [29]. This pleiotropic nature often leads as well to cytokine redundancy, wherein multiple cytokines have similar effects on a single cell type. For example, IL-4, IL-5, and IL-13 have similar activities to induce the differentiation of Th2 cells [28]. Some of the redundant nature of cytokines and their signaling events are due to the homology within the protein sequences or the use of the same or similar receptor types, each generating the same signaling pathways upon cytokine binding. However, other cytokines share minimal sequence homology but still result in similar downstream signaling events, possibly due to converging signaling pathways or differential distribution of cellular receptors on the target cells [30].

A number of approved disease treatments actually work via specific cytokine pathways, either targeting cytokines directly for activation/blockade or through the use of recombinant cytokines in an effort to create a specific response within the patient. These treatments include growth factors like epithelial growth factor receptor (EGFR) which is overexpressed in many tumors [31], tumor necrosis factor alpha (TNF*α*) which is a key proinflammatory protein in many autoimmune diseases [32], interferon-alpha (IFN*α*) which is used to treat chronic viral hepatitis [33], and AIDS-related Kaposi's Sarcoma [34], to name a few. Because of the complexity of cytokine biology, single cytokine analysis and evaluation is becoming limiting for its ability to diagnose, evaluate, and treat diseases, requiring more multiplex cytokine platforms to be used to consider the complexity of disease involving multiple cytokine perturbations.

## 2. Biomarker Discovery in Asthma

Typically during an asthmatic response, inflammation in the airway epithelium plays a central role in disease symptoms and progression. This lining acts as the primary interface between host and any environmental stimulus causing the allergic hyperresponsiveness, be it from microbes, particles, pollutants, and oxidants. These environmental insults trigger pathogen recognition receptors and/or already generated specific antibodies, stimulating the production of inflammatory cytokines and chemokines. Pollutants and oxidants on the other hand generally stimulate an inflammatory response via their direct impact on cells or the function of proteins, lipids, and nucleic acids, together resulting in cellular stress and injury [35–38]. In patients with asthma, persistent inflammatory stimuli modulate the functions of the airway epithelium, with both the innate and adaptive immune systems, further augmenting the development of a chronic allergic response. Once advanced enough, bronchial hyperreactivity (BHR) (the tendency of smooth muscle cells in people with asthma to react to nonspecific stimuli such as cold air and exercise), mucus overproduction, airway wall remodeling, and airway narrowing develop over the course of chronic exposure [39].

The immunohistopathologic features of asthma include epithelial injury and the infiltration of inflammatory cells, generally the eosinophils, mast cells, and lymphocytes into the lung. The adaptive immune response that develops is typically polarized to a Th2 CD4^+^ T cell response, characterized by elevated expression of IL-4, IL-8, and IL-10. This T cell polarization preferentially promotes B cell activation and antibody production favoring the allergic IgE and IgG isotypes. The resulting elevation in serum IgE and corresponding increases in infiltrating eosinophils, basophils, and mast cells are all prototypical hallmarks of atopic asthmatics [3, 39]. However, since heightened serum IgE levels are also seen in nonasthma related hypersensitivities, sera IgE levels may not be a suitable asthma biomarker when considered alone [40]. Therefore, it is necessary to look for surrogate biomarkers of this asthma endotype. As outlined in Table 1, cytokines released by the epithelium and the populations and numbers of activated inflammatory cells are of most interest (in terms of biomarkers) for asthma studies, because they recruit inflammatory cells to the airways and alter the activation state of airway resident cells [41].

Data collected from people with asthma and also in mouse models of asthma driven by inhalation of the model antigen ovalbumin (OVA) have shown that IL-4, IL-5, and IL-13 have potential to serve as an allergic asthma biomarker profile. Pulmonary Th2 polarization has long been considered as a typical characteristic of allergic asthma, which has been supported by studies with bronchoalveolar lavage (BAL) fluid, bronchial biopsies, and sputum from asthmatic patients [42–45]. Patients with mild-to-moderate asthma present with elevated levels of IL-4 and IL-5 in BAL fluid, and pulmonary isolates found elevated numbers of CD4+ T cells and a high degree of airway eosinophilia [46]. Careful enumeration of the levels of IL-4, IL-5, and IL-13 together and the levels of eosinophils in serum and tissue can classify asthma endotypes into Th2^hi^ and Th2^lo^ subsets [40, 47].

Mouse models of asthma support the roles of IL-4, IL-5, and IL-13 in disease development, as knockout mice for these cytokines show reduction in overall asthma symptoms [48]. In mice, IL-4 is necessary for the development of Th2 CD4 T cell response, the resulting IgG1 and IgE antibodies to OVA (the allergen stimulus), and also for priming the vessel wall for eosinophil extravasation [48, 49]. IL-4 also results in the priming and activation of basophils and mast cells, resulting in further release of inflammatory histamines and leukotrienes, further exacerbating and extending the chronic disease.

IL-13 has been reported to play an indispensable role in mounting bronchial hyperreactivity (BHR) and goblet cell hyperplasia, caused by elevated expression of the mucin proteins MUC5AC and MUC5B, leading ultimately to mucus overproduction and airway obstruction [50, 51]. In moderate-to-severe asthmatic patients with a high level of hematic eosinophilia, treatment with a monoclonal therapy that modulates the signaling of IL-4 receptor (dupilumab; Regeneron Pharmaceuticals), lung function was dramatically improved. With this treatment, the frequency of atopic dermatitis exacerbations was also reduced significantly, so that the drug received Breakthrough Therapy Designation from the FDA in 2014 [52]. Similar effects were seen from lebrikizumab (Genentech), an antibody blocking IL-13 in humans with Th2^hi^ endotype asthma [53]. Lastly, IL-5 promotes trafficking and maintenance of eosinophils within the lung tissue by directly recruiting eosinophils from the circulation and also promoting eosinophil differentiation from the bone marrow. A humanized monoclonal antibody that blocks IL-5 from signaling its receptor (mepolizumab; GlaxoSmithKline) has completed Phase III trials and is awaiting FDA and European clearance for patients with severe eosinophilic asthma [54]. When IL-5 was blocked, improvements in airway remodeling were seen and this improvement occurred alongside decreased eosinophils accumulation, further implying an exacerbating role for both IL-5 and the levels of eosinophils in the lungs [55].

Eosinophil induces BHR directly through eosinophil peroxidase and the resulting increases in inflammatory chloride and bromide ions, both significantly affecting airway remodeling during allergen exposures. Together with the antigen presenting nature of dendritic cells, the adaptive immune response is primed to respond to repeat antigen insult and airway hyperreactivity ensues. Eosinophil knockout mouse models of asthma have confirmed this cell population's role in contributing to airway wall remodeling and subepithelial membrane thickening [56, 57]. Eosinophilia in lung tissue is driven partially by the recruitment of eosinophils to the lung mucosa and interstitium due to airway produced IL-5, as well as production of eotaxin (CCL11), eotaxin-2 (CCL24), and eotaxin-3 (CCL26) [58, 59]. Eotaxins are CC chemokine with high specificity for eosinophils populations given this cell types preferential expression of eotaxin receptors, CCR2, CCR3, and CCR5. Eotaxins were first discovered from the purification of BAL fluid taken from allergen-challenged, sensitized guinea pigs, and, after their discovery, they were quickly identified in mouse and humans as well [60, 61].

In animal models of pulmonary allergic inflammation, eosinophil accumulation correlates with local eotaxin generation and differentiation, increased recruitment of eosinophils from circulation, and the rapid proliferation of eosinophils within the bone marrow [62, 63]. Increased expression of eotaxin at sites of allergic inflammation has been observed in both atopic asthmatics and nonatopic asthmatics and is also seen at increased levels in patient sputum [64–67]. Even in occupational asthmatics, increased eotaxin mRNA levels have been reported in sputum leukocytes, and Nakamura et al. have reported a correlation between eotaxin levels and compromised lung function in asthmatic patients [68, 69]. Similarly, patients with acute asthma are reported to display significantly higher plasma eotaxin levels compared to those with stable asthma [70].

There are also two functional homologues of eotaxin, eotaxin-2, and eotaxin-3, which show 40% homology to eotaxin at the amino acid level and also possess similar eosinophil-selective properties via CCR3 signaling [71, 72]. In assays of eosinophil chemotaxis, eotaxin and eotaxin-2 exhibit similar potencies in their ability to recruit eosinophils. Increased eotaxin-2 mRNA levels have been reported in bronchial biopsies taken from atopic and nonatopic asthmatics [65]. The chemotactic role of eotaxin-3 is less clear as there are contradictory results between various studies. In one investigation, eotaxin-3 shows only around 10% of the activity as eotaxin [72]; however, a more recent study by Provost et al. found that eotaxin-3 is a more effective chemoattractant than eotaxin and eotaxin-2 for eosinophils, specifically in asthmatics when compared to healthy volunteers [73]. Individually or collectively, these eosinophil chemokines could prove to be potential biomarkers for diagnosis and prognosis of asthmatic featured with eosinophilia, especially if eotaxin profiles between asthmatic patient groups can be identified.

Allergen induced expression of monocyte chemoattractant protein-4 (MCP-4; CCL-1) within asthmatic airways has also been shown recently as a potential biomarker [74]. MCP-4 signals through several receptors, including CCR2, CCR-3, and CCR5 and is a potent chemoattractant for asthma related cell populations including eosinophils, monocytes, and basophils [65, 75, 76]. MCP-4 signaling induces the release of histamine from IL-3-primed basophils and activates eosinophil respiratory burst. During allergic inflammation, MCP-4 levels are significantly elevated in asthmatic sputum, BAL fluid, and respiratory epithelium and within the cells that line the airways [77, 78]. Following allergen induced challenge, TNF*α* and IL-1*β* trigger the release of MCP-4 from the epithelium and endothelial cells that are inflamed, which in turn facilitates the recruitment of other types of asthma inducing inflammatory cells and the cytokines they produce.

The exacerbating role of MCP-4 expression in asthma has recently been supported by a large scale asthma study. Using 240 normal subjects, 356 chronic-stable asthma patients, and 30 patients who required emergency asthma treatments, the study found that plasma levels of MCP-4 in chronic-stable asthma patients compared to normal patients were significantly higher (399 pg/mL versus 307 pg/mL) [74]. Additionally, MCP-4 levels > 218 pg/mL marked a patient group at increased risk of developing asthma (*P* < 0.001 odds ratio). In acute asthma patients, MCP-4 levels were even higher during an exacerbation event when compared to chronic but stable asthma sufferers (513 versus 355 pg/mL). In conclusion, systemic levels of MCP-4 could prove an excellent asthma biomarker that can predict susceptibility to asthma, the severity of asthma exacerbations, and therefore potentially the efficacy of asthma control medicines.

Another potent cytokine which is released by inflamed epithelium and has been linked with asthma exacerbations is thymic stromal lymphopoietin (TSLP). TSLP is required to mount a normal CD4 T cell response where it acts directly on naive, but not, memory CD4 T cells, promoting their proliferation in response to cognate antigen [79]. However, TSLP's interaction with dendritic cell populations favors the polarization of primed CD4 T cells into Th2 phenotype [80, 81]. TSLP induces key changes in dendritic cells, such that, during antigen ligation to cognate T cells, DCs also are promoted to express the chemokine ligands 17 (CCL17) and CCL22, as well as inducing surface expression of OX40 12 ligand [81, 82]. In concert with other epithelial-derived inflammatory cytokines, such as TNF*α* and IL-1*β*, TSLP also activates mast cells which in turn can produce cytokines that increase the preferentially progression of Th2 polarization [41]. In mice genetically lacking the TSLP receptor, Th1 responses are elevated, including cytokine increases in levels of interleukin- (IL-) 12, interferon-*γ*, and immunoglobulin G2a (IgG2a), and also show lower expression of Th2 cytokines like IL-4, IL-5, IL-10, and IL-13. Furthermore, TSLPR KO mice challenged with inhaled antigen exhibit reduced pulmonary inflammation, a result that could be restored if wild type CD4 T cells were added back [80]. Reversion of pulmonary inflammation suggests that TSLP also can act directly on CD4 T cells during an inflammatory response. Collectively TSLP seems to play an important role in the development of allergic airway responses, and monitoring its levels in patient samples could provide another disease biomarker [83].

While IgE levels in atopic asthmatics can be a valuable disease indicator, IL-25 and IL-33 may be better surrogate biomarkers of both asthmatic disease and even disease stratification. In response to airway injury or allergen stimulation, epithelial cells can produce IL-25 and IL-33, which in turn can act on nearby innate lymphoid cells [84]. The innate populations receive these signals and can initiate degranulation of their cytokine and chemokine loaded granules, releasing additional allergen exacerbating factors. These cytokines include TSLP, IL-4, IL-5, and IL-9, all classical Th2 polarizing cytokines, and all cytokines that significantly affect the development of pulmonary inflammation. Interestingly, this same population is capable of producing these same Th2 cytokines (IL-5, IL-9, and IL-13) in response to mice infected with helminthes, promoting an eosinophilic gut response and enhanced mucus production [85]. These derived innate lymphoid cells resemble Th2 polarized CD4 T cells but do not express an antigen specific receptor [86–88]. It may be these cells that are responsible for the ability of RAG KO mice (i.e., mice lacking T and B cells) to generate a potent Th2-like allergic response and eosinophilia independent of any adaptive immune response. Data suggests that these cells originally develop from common lymphoid progenitors, and, following their initial allergen exposure, they can rapidly be activated to induce the features of atopic asthma following antigen reexposure [89].

IL-9 has also been recently evaluated for its use as an asthma biomarker given its heightened expression in the lungs of asthmatic patients. IL-9 functions in multiple potential asthmatic roles, from promoting mast cell growth and development to initiating IL-4 expression and subsequent B cell production of allergic antibodies [90, 91]. Mouse models of asthma have highlighted the role of IL-9, as treatment of asthmatic mice with neutralizing antibodies to IL-9 significantly alleviates symptoms [92, 93]. This blockade treatment also reduced the levels of airway remodeling which occurs during chronic asthma and also reduced the levels of infiltrating mast cells into the inflamed tissue [90]. Recent evidence suggests that IL-9 may arise from the aforementioned innate lymphoid cell population, and not directly from T cells [94, 95]. However, the contribution(s) of IL-9 to asthmatic disease, either from the innate or adaptive cell populations, remains to be determined. While the alleviation seen in mouse models of anti-IL-9 treatment provides a glimpse of promise for potential treatments and disease monitoring, this therapeutic effect has not yet been fully evaluated in asthmatic patients [96].

Many other novel biomarkers for asthma are potentially available from the current literature, as well as ongoing studies. For example, CCL-17 (TARC) is a dendritic and epithelial cell derived cytokine that is initially released upon allergen contact. CCL-17 is a potent chemoattractant for CCR4 expressing Th2 cells which then feed forward into the allergen derived inflammatory response. Recent studies found that CCL-17 levels were significantly elevated in asthmatic patient sputum and also were elevated in serum samples from children suffering from asthma [97, 98]. Interestingly CCL-17 could also serve as an indicator of treatment efficacy, as its levels significantly decline following steroidal treatment [98].

Cytokine biomarkers therefore hold significant promise for their potential ability to predict, diagnose, and monitor asthmatic disease. Because of the underlying heterogeneity of the asthmatic phenotypes and the general complexity of cytokine biology, examination of a single biomarker, or even a small handful of molecules, to detect and/or treat asthmatic patients is unrealistic. With their ability to be quantified in noninvasive sample types, like serum, sputum, or even exhaled breath condensates, cytokines could complement current diagnostic and monitoring measures to give a more complete profile of patient disease. Combining these two methods of patient analysis, comparisons between healthy and diseased patients may better help stratify potential biomarkers to their respective disease states.

## 3. New Discovery in Asthma by Antibody Array Technology

### 3.1. Antibody Arrays Technology

Traditionally, the measurement of cytokine/protein mediators involves the use of bioassays, enzyme assays, or immunoassays. Since these are single target assays where simultaneous detection of other targets is not possible, the use of these assays requires a sizeable amount of sample and/or budget. Also, the investigator must already have a fairly specific hypothesized protein biomarker to evaluate. This makes true discovery of the unknown increasingly difficult. If the initially chosen markers are not differentially present in established sample groups (especially small sample groups), entire sample sets or experiments may be abandoned, all while some combination of these markers with others may remain undiscovered.

In the past twenty years, the discovery, characterization, and application of biomarkers in clinical and the basic research fields have exploded with a number of critical advancements and findings. Through the availability of DNA sequencing technologies, the completion of the Human Genome Project, and genetic sequencing platforms, the comprehensive and rapid detection of genes in a sample is now possible. High-throughput gene microarray technology has greatly expanded the ability to define nucleic acid biomarkers of disease within a single sample, and the same is now coming true with protein biomarkers. Protein microarrays have been adapted from DNA microarray platforms and now offer the ability to do multiplex protein biomarker discovery and to do so in a high-throughput manner. These protein arrays, also known as antibody arrays or antibody microarrays, are one of the most promising platforms to break through the single biomarker bottleneck that the field is currently stuck in.

**Figure 1:**
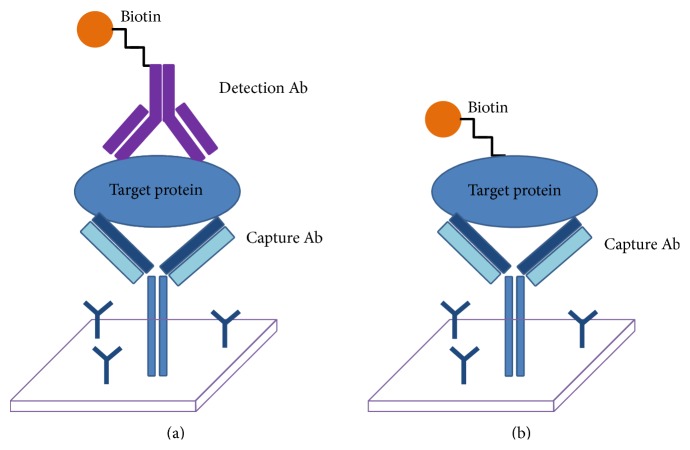
The principle employed in most antibody arrays. (a) The sandwich method which requires an immobilized capture antibody and a labeled detection antibody. (b) Direct-label method with capture antibody and labeled analyte.

The design principle of antibody arrays is usually based on either a sandwich based ELISA immunoassay or a direct-labeling of target proteins approach. Similar to common single target sandwich ELISA platforms, multiplex antibody array platforms utilize an antibody pair, where a capture antibody is immobilized on the surface of glass slides or nitrocellulose membranes and is paired with a labeled target specific detection antibody in solution (Figure 1(a)). This method has the advantages of excellent specificity and sensitivity given the dual binding requirements for target signal detection. The capture antibody is specific for one region of the target protein, while the detection antibody recognizes a different region of the target, combining to remove almost all potential for epitope cross-reactivity. However, the combinations and the number of protein targets to be measured in each array are limited because of cross-reactivity between detection antibodies within a single array. To overcome this restriction, multiple independent arrays with panels of nonoverlapping and cross-reactivity compatible antibody pairs can be employed to allow increasing numbers of cytokines to be measured [99].

Alternatively, label-based strategies can be employed to remove the need for a detection antibody and to dramatically increase the size of the potential array [100]. In the label based method, the sample itself is directly labeled with some substrate that can later be measured (biotin for example), while the capture antibody is still arrayed onto the solid surface (Figure 1(b)). In this case, there is an increase in sensitivity, as the dual binding requirement is removed, but there is a simultaneous loss in some of the specificity seen with a standard detection pair from a sandwich ELISA format. With only a single antibody required for target detection, label based techniques are especially useful for small peptides/molecules or for more novel proteins for which an antibody pair may not be available. Both of these platform types have been extensively published in the discovery and analysis of disease biomarkers and offer the largest simultaneous arrays available on the market [101, 102].

Glass slide based arrays provide several advantages in comparison with membrane based or 96-well based multiplex platforms. The capability to spot the target antibody with a diameter of <200 *μ*m allows glass based antibody arrays to be much smaller in size and require less sample for target detection, while also allowing them to be robotically printed in mass quantities suitable for high-throughput analysis [103]. Another advantage of the glass slide support format is the use of fluorescence as a signal readout. Fluorescence detection affords a much larger linear range of signal detection and superior signal stability over the long term when compared to chemiluminescent detection technologies [103–105]. This platform is being promoted and explored by a number of companies including RayBiotech, Abcam, Sigma-Aldrich, R&D Systems, Full Moon Biosystems, Gentel, Whatman, Aushon, MesoScale, and Quansys. These powerful platforms have been extensively published in recent years since their development in the early 2000s and have also been comprehensively applied in preclinical and basic research for biomarker discovery [106–109].

### 3.2. New Discovery of Asthma Biomarkers by Antibody Array Technology

The application of antibody arrays in biomarker discovery has allowed significant progress on a number of disease fronts, including cancer, neurodegeneration, cardiovascular disease, as well as in asthma. As previously described, the complex biology of asthma syndrome has linked a number of cytokines and chemokines as potential disease biomarkers. Additional asthma biomarker limitations are due to a number of single target cytokine biomarker studies that have been of limited use or yielded incomplete definition of disease after further evaluation. This is where multiplex analysis has such a broad appeal as it affords a large nonbiased look at large numbers of proteins within a sample and can include a broad array of protein factors. Published biomarkers that have been explored and/or discovered with antibody arrays have been partially compiled in Table 2. It is of note that antibody arrays have both confirmed the previously suggested biomarkers in a number of studies and also unveiled a number of novel biomarkers as well.

Recently, Patil et al. used antibody arrays to study the serological profiles from nonsmokers with moderate and severe persistent asthma to sera of nonasthmatic healthy controls. Using a 50-target protein multiplex array which included detection of cytokines, chemokines, and growth factors, this group found that several proteins could serve as potential disease biomarkers (notably, FGF, HGF, and SCGF*β*) for the moderate and severe asthma groups. Furthermore, these same biomarkers in addition to IL-18 were found to positively correlate with poor asthmatic control and were associated negatively with quality of life scores [110]. With the hopes of identifying critically important inflammatory molecules in COPD and asthma, Kim et al. analyzed asthma sputum samples with a 79 target multiplex array. Among the tested cytokines, asthmatic patient sputum displayed significantly elevated levels of CXCL1, eotaxin-2, and CCL18. Interestingly, the levels of CCL18 correlated significantly with the overall levels of eosinophils in the sputum sample, which is a significant marker of underlying disease [111]. This might allow for a noninvasive and novel diagnostic measurement of patient disease from a simple sputum sample and also further highlights a role of CCL18 in asthmatic disease.

Given the influx of inflammatory cells into the lungs during asthmatic exacerbations, noninvasive sputum samples could allow for biomarker detection of the underlying inflammation within the lungs. This hypothesis was tested by Hastie et al. using inflammatory antibody arrays in a cohort of asthma patients in the Severe Asthma Research Program. Sputum samples indicated that elevated levels of eosinophil and neutrophil counts were associated with decreased lung function and elevated cytokine levels of brain-derived neurotrophic factor, IL-1b, and macrophage inflammatory protein 3a/CCL20 were associated with increased disease severity and were also linked to the increased neutrophil counts [112]. Similarly Matsunaga et al. have also shown a strong correlation between RANTES (CCL5) levels and FEV_1_, as well as expression levels of TNF*α* and TGF*β*1 with BHR [113, 114]. In addition, the expression level of these cytokines was also positively and specifically correlated with the disease symptoms and disease severity in asthmatic patients.

A very unique study and sample type was recently undertaken by Nakamura et al. Using exhaled breath condensates (EBC), this group compared this novel sample type to the invasive BAL fluid samples taken from asthmatic patients [115]. They found that levels of cytokines in the EBC, including TNF-*α* and RANTES, significantly correlate with those presented in the patient's BAL fluid. BAL collection in patients is an extremely unpleasant and invasive procedure requiring the flushing of patient's lungs with fluid. However, EBC could afford a nontraumatic sample type that is much more easily obtained and also more economically viable for patient disease monitoring. The authors also noted a correlation of the levels of TNF-*α* and RANTES in EBC samples with the levels of lymphocytes eventually extracted from the BAL isolates. Lung lymphocyte counts are one of the critical indicators associated with asthmatic disease states, so EBC measurements might allow yet another option for diagnostic disease monitoring.

Along these same experimental lines, Matusunaga's group also used a large antibody array to monitor the cytokine profiles of asthmatic patients undergoing corticosteroid treatment [116]. Patients treated with corticosteroids showed decreased levels of CXCL10 and increased levels of IL-4 and RANTES compared to untreated controls, and these changes correlated to improved FEV_1_ and overall airway obstruction. These findings strongly suggest that IL-4 and RANTES levels in EBC could not only be applied as predictive biomarkers for the success of steroidal therapies but also mark cytokines with potential as therapeutic targets during disease intervention and monitoring.

Like applications in other diseases, inflammatory antibody arrays have also been explored during the evaluation of drugs for disease efficacy and other disease indicators. Antibody arrays have even been used to study homeopathic remedies against asthmatic disease, like in the case of the antiasthmatic effect of mangiferin. Mangiferin is a major bioactive ingredient in* Mangifera indica *Linn. leaves and is currently used as an indigenous remedy for respiratory diseases in traditional Chinese medicine. To study mangiferin's effects, the authors used a mouse allergen model (Ova model) to evaluate the levels of Th1/Th2 cytokine profiles during disease. Mangiferin treatment reduced the inflammatory cell influx of eosinophils, reduced the BAL fluid levels of PGD2, and reduced the levels of serum ovalbumin specific IgE [118]. Antibody array analysis confirmed the downregulation of Th2 related cytokines (IL-4, IL-5, and IL-13), and even the upregulation of Th1 promoting cytokines like IFN-*γ*, IL-2, and IL-12 during mangiferin treatment. These results suggest mangiferin's potential as an antiasthmatic therapy and might also suggest its potential route of action, since the treatment resulted in conversion of allergic Th2 response into a more nonallergenic Th1 type response. These inflammatory array panels, or even more broad panels, could be used to monitor other antiasthmatic drugs for disease outcome, progression, and efficacy.

While targeted inflammatory antibody arrays may seem to be the most obvious candidates for asthmatic disease analysis, other arrays also have shown promise in asthma biomarker studies. Several studies have shown that asthmatic airways have an increased number and size of vascular structures, which contribute to airflow obstruction and hyperresponsiveness [119–121]. Therefore evaluation of angiogenic markers in a multiplex array might prove of great utility in monitoring patient airway remodeling. Using this hypothesis, BAL samples from atopic asthma patients were found to contain significantly elevated levels of angiogenic growth factors including angiogenenin, VEGF, and MCP-1 when compared to nonatopic healthy controls. Healthy controls conversely showed no change in the levels of common antiangiogenic factors. This proangiogenic effect was confirmed in vitro when the same group found that BAL taken from asthmatic patients could support tubule vessel formation in a coculture of human endothelial and dermal fibroblasts. This vessel formation capability arose primarily due to the presence of VEGF in the BAL sample. This observation suggests that a panel of angiogenic markers, like angiogenin, VEGF, and MCP-1, could serve as a novel diagnostic panel to monitor vascular disease modeling and may be a good companion study to other traditional diagnostic measures [117].

## 4. Conclusion

Over the past few years there have been numerous publications providing a substantial body of information collectively revealing the multitude of biological pathways involved in the pathogenesis of asthma. More recently the heterogeneous nature of asthmatic disease has been identified and has suggested the presence of many disease endotypes that warrant special diagnostic measures and even specific treatments for the best disease outcomes. Multiple aspects of host immunity are involved in the development, exacerbations, and eventual chronic nature of asthma. The major reasons for allergic disease development include common allergens, environmental triggers, and even respiratory viruses. These sensitizing agents create specific disease outcomes, and our current understanding of the disease implies that each may need a specific disease treatment. Understanding these individual disease cohorts and their underlying causes is critical to making the best outcome for disease possible.

Diagnostic measures currently revolve around visual inspection and spirometry, but a growing field of research is finding that these two methodologies are insufficient for the best classification of asthmatic disease. There are extensive endeavors currently underway in discovering protein biomarkers that could be used in asthma diagnosis, prognosis, and serving as therapeutic targets. No single protein target is likely to be effective for all diseases, especially one of such complexity, but targeted cytokine panels may provide hope for the multiprotein detection panels needed for future basic research and clinical trials for this debilitating syndrome.

Cytokine antibody arrays have gained popularity over the past several years with hundreds of publications in scientific journals. Their popularity is owed to their ability to approach a sample in a nonbiased way and detect a wide range and large number of cytokines simultaneously. This broad view allows the best chance at discovering the vast network of complex interactions occurring in disease states. As more and more cytokines are evaluated for any disease state and more and more samples probed, there is no doubt that we can uncover complex and in-depth molecular pathways involved in disease outcomes, disease progression, and disease therapies and cures.

## Figures and Tables

**Table 1:** Cytokines as biomarkers in asthma explored in these years.

Cytokine targets	Typical physiologic functions	Potential application	Sample source
IL-4	Activating Th2 immunity; priming the vessel wall for eosinophil extravasations	Diagnosis, therapeutic	Tissue, sputum, BALF, serum
IL-5	Promoting eosinophilia	Diagnosis, therapeutic	Tissue, sputum, BALF, serum
IL-13	Mounting BHR, goblet cell metaplasia	Diagnosis, therapeutic	Tissue, sputum, BALF, serum
IL-25	Activating innate lymphoid cells	Diagnosis, therapeutic	Tissue, sputum, BALF, serum
IL-33	Activating innate lymphoid cells	Diagnosis, therapeutic	Tissue, sputum, BALF, serum
TSLP	Activating dendritic cells and promoting Th2 immunity	Diagnosis, therapeutic	Tissue, serum
Eotaxin-1/CCL11	Eosinophil chemotaxis	Diagnosis, prediction	Tissue, sputum, BALF
Eotaxin-2/CCL24	Eosinophil chemotaxis	Diagnosis	Tissue, sputum, BALF
Eotaxin-3/CCL26	Eosinophil chemotaxis	Diagnosis	Blood, tissue
MCP-4/CCL13	Chemoattractant for eosinophils, monocytes, lymphocytes, and basophils	Diagnosis, prediction	Sputum, BALF, plasma
IL-9	Promoting IL-4-driven antibody, inducing goblet cell metaplasia	Diagnosis, therapeutic	Tissue, serum
CCL17	Recruitment of Th2 cells	Diagnosis, prediction	Sputum, serum

**Table 2:** Cytokine biomarkers in asthma explored by using antibody array technology.

Authors [reference]	Cytokine panels	Potential application	Sample source
Patil et al. [110]	Interleukin-18, FGF, HGF, and SCGF*β*	Diagnosis, prediction	Serum
Kim et al. [111]	GRO*α*/CXCL1, Eotaxin-2/CCL24, and PARC/CCL18	Diagnosis, therapeutic	Sputum
Hastie et al. [112]	BDNF, IL-1b, and MCP 3a/CCL20	Prediction	Sputum
Matsunaga et al. [113, 114]	RANTES/CCL5, TNF*α* and TGF*β*1	Therapeutic evaluation	Exhaled breath condensate (EBC)
Nakamura et al. [115]	TNF-*α* and RANTES	Diagnosis	BALF
Matsunaga et al. [116]	IL-4 and RANTES	Diagnosis, prediction	Exhaled breath condensate (EBC)
Simcock et al. [117]	Angiogenin, VEGF, and MCP-1	Diagnosis, therapeutic	BALF

## Abbreviations

AIDS: Acquired immunodeficiency syndrome
AD: Alzheimer's disease
BAL: Bronchoalveolar lavage fluid
BHR: Bronchial hyperreactivity
CCL: CC chemokine ligand
CRP: C reactive protein
CSF: Cerebrospinal fluid
CT-1: Cardiotrophin-1
CXCL: CXC chemokine ligand
EBC: Exhaled breath condensate
EGFR: Epithelial growth factor receptor
ELISA: Enzyme-linked immunosorbent assay
FEV_1_: Forced expiratory volume in 1 second
FGF: Fibroblast growth factor
GRO: Growth-regulated oncogene
HGF: Hepatocyte growth factor
IBD: Inflammatory bowel disease
IFN: Interferon
IL: Interleukin
IL-2R*α*: Interleukin 2 receptor alpha
ILCs: Innate lymphoid cells
MAb: Monoclonal antibody
MCP-1: Monocyte chemotactic protein 1
MCP-4: Monocyte chemotactic protein 4
OVA: Ovalbumin
PARC: Pulmonary and activation-regulated chemokine
RA: Rheumatoid arthritis
RANTES: Regulated upon activation normal T cell expressed and secreted
SCGF*β*: Stem cell growth factor beta
TGF*β*1: Transforming growth factor beta-1
Th1: CD4^+^ type 1 helper T cells
Th2: CD4^+^ type 2 helper T cells
TLR: Toll-like receptor
TNF: Tumor necrosis factor
TSLP: Thymic stromal lymphopoietin
VEGF: Vascular endothelial growth factor.
